# An Atypical Presentation of Heroin Inhalation Induced Leukoencephalopathy (Chasing the Dragon)

**DOI:** 10.7759/cureus.11215

**Published:** 2020-10-28

**Authors:** Muhammad Atif Masood Noori, Sherif Elkattawy, Islam Younes, Ramez Alyacoub, Dhaval Desai

**Affiliations:** 1 Internal Medicine, Dow Medical College, Karachi, PAK; 2 Internal Medicine, Rutgers New Jersey Medical School/ Trinitas Regional Medical Center, Elizabeth, USA; 3 Cardiovascular Disease, Jersey Shore University Medical Center, Neptune, USA

**Keywords:** chasing the dragon, heroine inhalation, leukoencephalopathy, heroine abuse, spongiform degeneration, antioxidants

## Abstract

Heroin leukoencephalopathy is associated with 'Chasing the dragon,' which is a heroin vapour inhalation method that is different from smoking or sniffing heroin. The clinical presentation ranges from mild to severe disease. Mild disease is characterized by inattentiveness and ataxia. In moderate diseases, extrapyramidal symptoms predominate, and finally, severe disease is characterized by generalized motor impairment, with death occurring in two-third of cases. We now report a rare presentation of the disease in a 60-year-old female with a past medical history of heroin abuse who presented to ED with signs and symptoms of confusion and restlessness. MRI brain without contrast showed diffuse symmetric increased intensity signals throughout the white matter. Electroencephalogram (EEG) revealed mild diffuse slowing with no lateralization. The patient was started on Vitamin E and was transferred to a rehab facility with following up neurology as an outpatient.

## Introduction

Leukoencephalopathy, due to heroin inhalation colloquially known as 'chasing the dragon,' is a rare complication of heroin abuse. 'Chasing the dragon' a term derived from the method of inhalation of heroin fumes that is different from sniffing or smoking heroin [1]. A small amount of heroin powder is heated in aluminum foil, which then releases a white smoke resembling a dragon tail. The vapors released are chased or inhaled through a small tube or straw [2]. The result includes an aggressive, toxic leukoencephalopathy with pathognomonic neuropathologic features in addition to hydrocephalus and movement disorders. The clinical severity ranges from mild to moderate to severe. Mild cases survive with minor sequelae, while severe presentations can progress to death [3]. We report a fascinating case featuring an atypical presentation of leukoencephalopathy due to heroin inhalation.

## Case presentation

A 60-year-old Caucasian female with the unknown past medical history presented to the emergency department via her sister for evaluation of altered mental status. The patient was a poor historian, and most of the history was obtained from the patient's sister. As per collateral, the patient had been behaving "differently" for the past month. The patient was unaware of why she was in the hospital. She replied, 'does not know' to almost every question. She denied fever, headache, dizziness, nausea, vomiting, muscle weakness, sensory deficit, gait abnormality, hallucination, homicidal or suicidal ideation. She also denied smoking or any illicit drug use; however, her sister reported the patient has been inhaling heated vapours of heroin for an unknown duration.

On examination, she looked confused and restless, was alert and oriented to person and place but not to time, and intermittently laughing throughout the interview. CNS examination revealed normal strength and sensation in all extremities, including proprioception. No gait abnormalities or nystagmus was observed.

**Figure 1:**
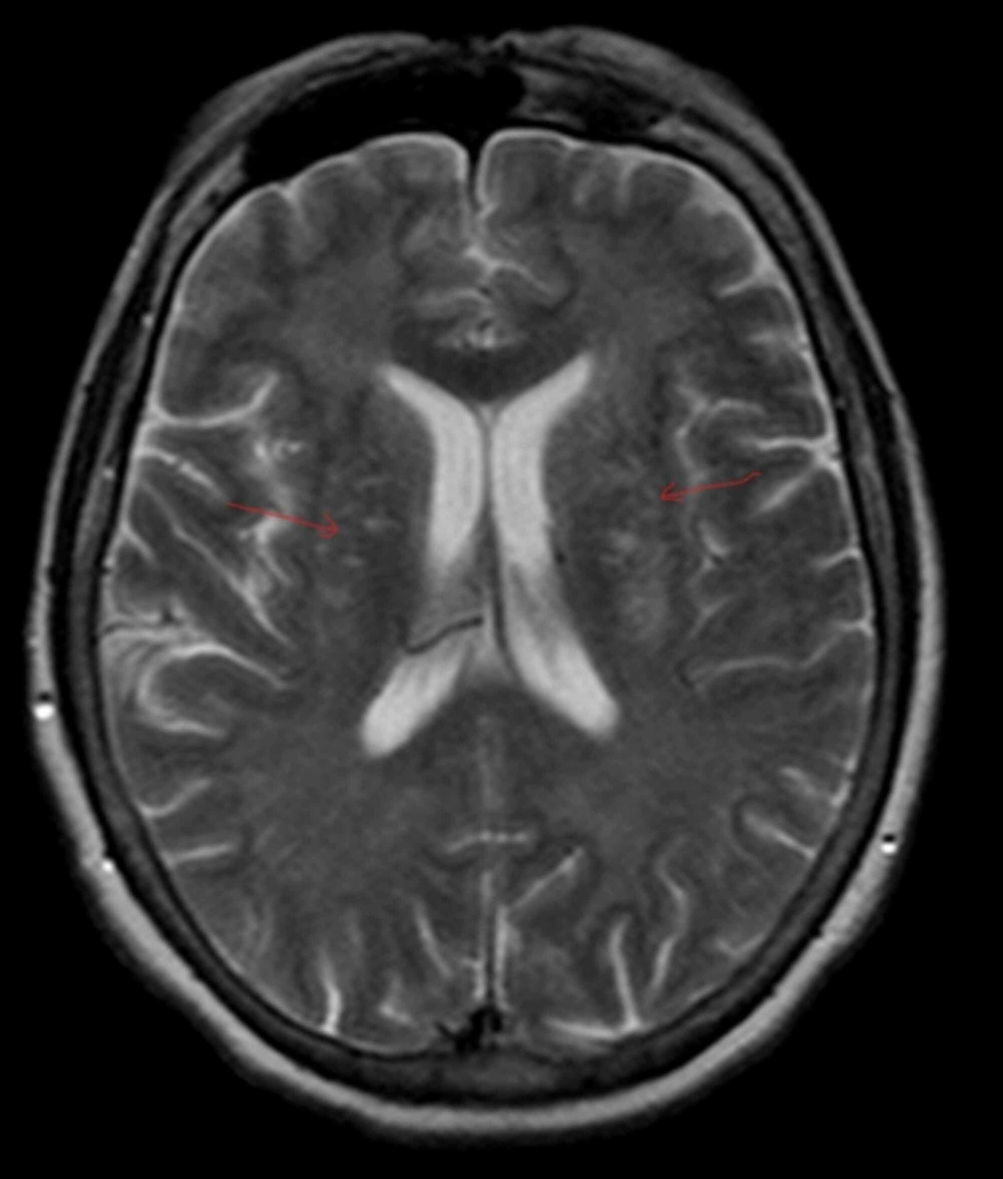
T2 weighted Axial MRI of the brain showing diffuse symmetric white matter hyperintensities in different parts of the brain

**Figure 2:**
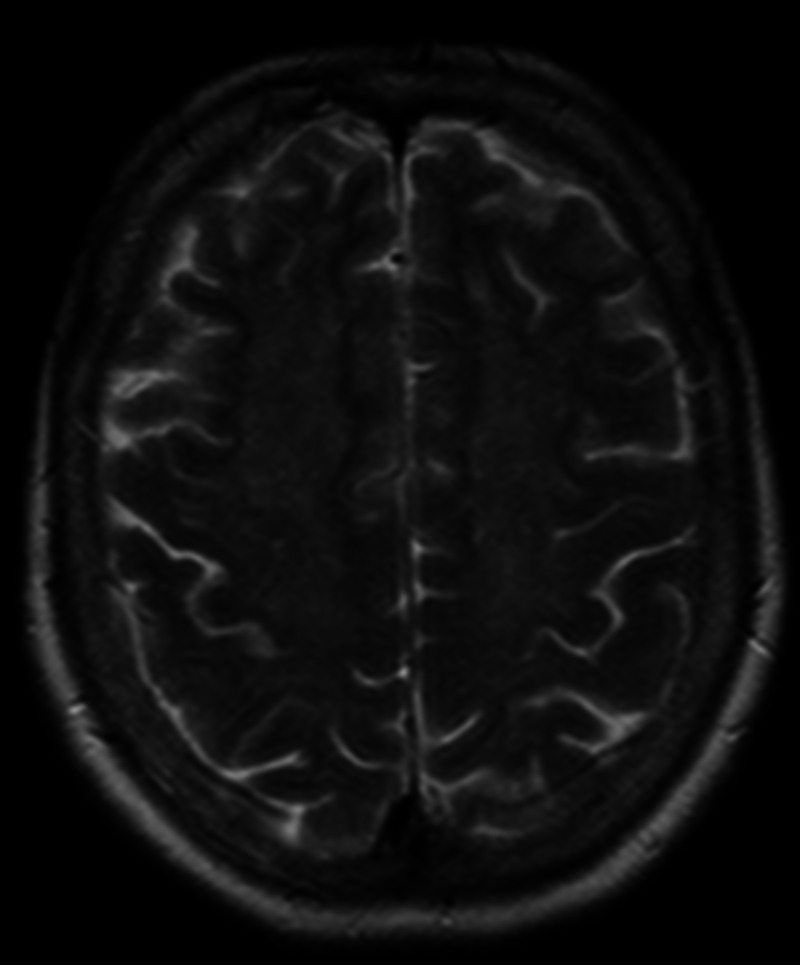
T2 weighted Axial MRI of the brain showing diffuse symmetric increased intensity signals throughout the white matter in frontal and parietal lobes

Laboratory investigations done on admission, including complete blood count and metabolic panel, were unremarkable; WBC 8.3 k/ul, Hb 13.3 g/dl, platelets 392 k/ul, creatinine (Cr) 0.7 mg/dl, sodium (Na) 135 mmol/l, potassium (K) 4.8 mmol/l. The urine drug screen was negative. The CT head was unremarkable. MRI brain without contrast showed diffuse symmetric increased intensity signals throughout the white matter (Figures 1 and 2). Lumbar puncture was also done, which was unremarkable. EEG revealed mild diffuse slowing with no lateralization.

The patient was started on Vitamin E 400 IU twice daily and was transferred to a rehab facility with following up neurology as an outpatient. She was counselled regarding abstaining from heroin.

## Discussion

Heroin inhalation causing leukoencephalopathy was first initially described in a report from the Netherlands [1]. Inhaled heroin has become increasingly popular to avoid the risk of diseases associated with parenteral administration of heroin Heroin and its metabolite cross the blood-brain barrier after inhalation. However, the exact mechanism of causing neuronal injury is unknown [2].

The clinical presentation ranges from mild to severe disease. Mild disease is characterized by inattentiveness, ataxia, and confusion. In moderate diseases, extrapyramidal symptoms predominate, and finally, severe disease is characterized by generalized motor impairment with death occurring in two-thirds of cases [3,4]. In addition, various features of Parkinsonism and hydrocephalus, later requiring surgical intervention, have been described as a complication in case reports [1,5]. Our patient had no symptoms described above, except for the fact that she was confused and restless.

Characteristic MRI findings include symmetrically increased T2 and T2-FLAIR signal intensity in the cerebellum, posterior cerebrum, and posterior limbs of the internal capsule. Grey matter affected in anoxic encephalopathy is usually spared [6]. EEG usually shows diffuse slowing without epileptiform activity [7]. Brain biopsy findings include spongiform degeneration of white matter and vacuole formation in oligodendroglia and myelin sheath. The diagnosis is clinical and should be suspected in patients with a history of chasing the dragon presenting with neurobehavioural symptoms and/or cerebellar, pyramidal, or extrapyramidal signs, supported by characteristic neuroimaging features.

There is no established treatment for heroin leukoencephalopathy. Kriegstein AR et al. reported clinical improvement in patients treated with antioxidants, including coenzyme Q [7]. This clinical improvement with antioxidant therapy observed in a few patients indicates mitochondrial dysfunction in the development of the disease. Antioxidant therapy with coenzyme Q10, Vitamin C and E is suggested, considering the absence of proven treatment and low side effect profile.

## Conclusions

Heroin induced leukoencephalopathy is a rare complication of heroin abuse. Diagnosis should be considered in a patient with a history of 'chasing the dragon' and neurobehavioral changes. Our patient presented only with mild symptoms of confusion and restlessness. Clinical features with a history of heroin use, along with MRI findings, helped establish the diagnosis and starting treatment.
